# Supplementary material for: Cenozoic history of the tropical marine biodiversity hotspot
Source: Nature. 2024 Jun 26;632(8024):343–9. doi: 10.1038/s41586-024-07617-4 (PMC11306107; doi:10.1038/s41586-024-07617-4)
Supplement: Supplementary file 1 — Supplementary Tables 1–7. [file 41586_2024_7617_MOESM1_ESM.docx]

**Supplementary Information for “Cenozoic history of the tropical marine biodiversity hotspot”**

**Authors:**

Skye Yunshu Tian^1,2^*,**

Moriaki Yasuhara^2,3^*,**

Fabien L. Condamine^4^**

Huai-Hsuan M. Huang^5^

Allan Gil S. Fernando^6^

Yolanda M. Aguilar^7^

Hita Pandita^8^

Toshiaki Irizuki^9^

Hokuto Iwatani^10^

Caren P. Shin^11,12^

Willem Renema^13,14^

Tomoki Kase^15^

^1^Steinmann-Institut für Geologie, Mineralogie und Paläontologie, Universität Bonn, Bonn, Germany

^2^School of Biological Sciences, Area of Ecology and Biodiversity, Swire Institute of Marine Science, Institute for Climate and Carbon Neutrality, and Musketeers Foundation Institute of Data Science, The University of Hong Kong, Hong Kong SAR

^3^State Key Laboratory of Marine Pollution, City University of Hong Kong, Hong Kong SAR

^4^CNRS, Institut des Sciences de l’Evolution de Montpellier, Université de Montpellier, Place Eugène Bataillon, 34095 Montpellier, France

^5^Department of Geosciences, Princeton University, New Jersey 08544, USA

^6^National Institute of Geological Sciences, University of the Philippines, Diliman, Quezon City 1101, Philippines

^7^Marine Geological Survey, Mines and Geosciences Bureau, North Avenue, Diliman, Quezon City 1101, Philippines

^8^Department of Geological Engineering, Faculty of Mineral Technology, Institute Teknologi Nasional Yogyakarta, Yogyakarta 55281, Indonesia

^9^Department of Geoscience, Interdisciplinary Graduate School of Science and Engineering, Shimane University, 1060 Nishikawatsu-cho, Matsue 690-8504, Japan

^10^Division of Earth Science, Graduate School of Sciences and Technology for Innovation, Yamaguchi University, Yoshida 1677-1, Yamaguchi 753-8511, Japan

^11^Paleontological Research Institution, 1259 Trumansburg Road, Ithaca, New York 14850, USA

^12^Department of Earth and Atmospheric Sciences, Cornell University, 112 Hollister Drive, New York 14850, USA

^13^Naturalis Biodiversity Center, Darwinweg 2, 2333 CR Leiden, the Netherlands

^14^IBED, University of Amsterdam, Sciencepark 904, 1098 XH Amsterdam, the Netherlands

^15^National Museum of Nature and Science, Department of Geology and Paleon-

tology, Tsukuba, Ibaraki 305-0005, Japan

*Correspondence to: [skyeystian@gmail.com](mailto:skyeystian@gmail.com); [moriakiyasuhara@gmail.com](mailto:moriakiyasuhara@gmail.com)

**Equal contribution

**Supplementary table 1**. **Predicted species richness at n=200 individuals from rarefaction for all the Cenozoic IAA samples.**

| Sample | Island | Age (Ma) | Interval | E(S200) |
| --- | --- | --- | --- | --- |
| 06KW4B | Java | 39 | Eocene | 11.4326 |
| 06KW06 | Java | 39 | Eocene | 7.44008 |
| 06KW05 | Java | 39 | Eocene | 12.75901 |
| 06BA18C | Java | 18.9 | early Miocene | 22.24207 |
| 06BA18A | Java | 18.9 | early Miocene | 21.72184 |
| 06BA18B | Java | 18.9 | early Miocene | 19.62781 |
| 06BA18D | Java | 18.9 | early Miocene | 21.68406 |
| 06BA18 | Java | 18.9 | early Miocene | 24.60658 |
| CDN12 | Negros | 15.51 | middle Miocene | 31.57105 |
| CDN15 | Negros | 15.51 | middle Miocene | 16.00905 |
| CDN18-5 | Negros | 15.51 | middle Miocene | 30.10206 |
| MYA1-2 | Cebu | 14.23 | middle Miocene | 13.71209 |
| MYA1-3 | Cebu | 14.23 | middle Miocene | 8.884903 |
| CDN3-3 | Negros | 13.82 | middle Miocene | 18.42897 |
| MYA1-4 | Cebu | 12.17 | middle Miocene | 10.55018 |
| MYA1-5 | Cebu | 10.73 | late Miocene | 23.27706 |
| DGH1-3 | Bohol | 10.09 | late Miocene | 54.20934 |
| MYA1-6 | Cebu | 10.09 | late Miocene | 27.37713 |
| MYA1-7 | Cebu | 10.09 | late Miocene | 19.3965 |
| TF110-SR42 | Kalimantan | 9.6 | late Miocene | 20.05962 |
| TF110-WR18 | Kalimantan | 9.6 | late Miocene | 15.83066 |
| TF517-FW03 | Kalimantan | 9.1 | late Miocene | 23.56135 |
| TF534-NS2 | Kalimantan | 9.1 | late Miocene | 18.89891 |
| CDN18-6 | Negros | 8.49 | late Miocene | 16.25194 |
| CDN8-1 | Negros | 8.49 | late Miocene | 31.68977 |
| CDN8-2 | Negros | 8.49 | late Miocene | 16.93883 |
| TCN2 | Panay | 8.49 | late Miocene | 30.12848 |
| CDN10 | Negros | 7.625 | late Miocene | 48.60222 |
| CDN11 | Negros | 7.625 | late Miocene | 42.83859 |
| CDN19-8 | Negros | 7.625 | late Miocene | 9.131757 |
| CDN5 | Negros | 7.625 | late Miocene | 50.31631 |
| CDN7-1 | Negros | 7.625 | late Miocene | 53.86478 |
| CAB14 | Luzon | 6.91 | late Miocene | 13.91007 |
| CAB16 | Luzon | 6.91 | late Miocene | 12.34517 |
| CAB3 | Luzon | 6.91 | late Miocene | 42.35302 |
| CAB8 | Luzon | 6.91 | late Miocene | 31.72442 |
| TF168-WR | Kalimantan | 6.5 | late Miocene | 13.43023 |
| PSS1(2) | Panay | 6.475 | late Miocene | 31.24528 |
| TCN5 | Panay | 6.07 | late Miocene | 38.52748 |
| B1 | Cebu | 5.47 | late Miocene | 42.46874 |
| B2 | Cebu | 5.47 | late Miocene | 45.96162 |
| B3 | Cebu | 5.47 | late Miocene | 40.7101 |
| B4 | Cebu | 5.47 | late Miocene | 32.10781 |
| B5 | Cebu | 5.47 | late Miocene | 42.39228 |
| B6 | Cebu | 5.47 | late Miocene | 39.08555 |
| B7 | Cebu | 5.47 | Late Miocene | 45.45859 |
| B8 | Cebu | 5.47 | late Miocene | 42.18706 |
| B9 | Cebu | 5.47 | late Miocene | 49.13405 |
| BGH1-2 | Cebu | 5.47 | late Miocene | 49.42383 |
| BGH1-3 | Cebu | 5.47 | late Miocene | 50.65604 |
| BGH1-4 | Cebu | 5.47 | late Miocene | 54.99999 |
| BGH1-5 | Cebu | 5.47 | late Miocene | 37.54997 |
| BGH1-6 | Cebu | 5.47 | late Miocene | 38.8855 |
| SID2 | Bohol | 5.47 | late Miocene | 56.57452 |
| SID2' | Bohol | 5.47 | late Miocene | 53.4719 |
| SID3 | Bohol | 5.47 | late Miocene | 16.52972 |
| SID4 | Bohol | 5.47 | late Miocene | 36.80631 |
| SID5 | Bohol | 5.47 | late Miocene | 8.003911 |
| SID7 | Bohol | 5.47 | late Miocene | 11.54038 |
| SID8 | Bohol | 5.47 | late Miocene | 20.16544 |
| CJR07 | Java | 4.465 | Pliocene | 6.011276 |
| MNT11 | Java | 4.465 | Pliocene | 26.03531 |
| SDE4 | Java | 4.465 | Pliocene | 54.54362 |
| SDE6 | Java | 4.465 | Pliocene | 75.50581 |
| SDE2 | Java | 3.965 | Pliocene | 25.43793 |
| S164 | Leyte | 3.935 | Pliocene | 51.21955 |
| S203 | Leyte | 3.89 | Pliocene | 85.40195 |
| S724 | Leyte | 3.89 | Pliocene | 82.27782 |
| BYH1A | Java | 3.875 | Pliocene | 46.73016 |
| BYH1B' | Java | 3.875 | Pliocene | 40.91881 |
| BYH1B'' | Java | 3.875 | Pliocene | 36.54091 |
| MNT5 | Java | 3.875 | Pliocene | 54.4615 |
| MNT6 | Java | 3.875 | Pliocene | 59.14764 |
| MNT7 | Java | 3.875 | Pliocene | 60.12091 |
| TinagoA | Leyte | 3.875 | Pliocene | 79.88216 |
| TinagoB | Leyte | 3.875 | Pliocene | 65.14578 |
| S723 | Leyte | 3.835 | Pliocene | 72.83287 |
| LLB | Leyte | 3.775 | Pliocene | 81.78283 |
| S-120 | Leyte | 3.23 | Pliocene | 86.13071 |
| TKS1-1 | Java | 2.93 | Pliocene | 42.58395 |
| TKS1-2 | Java | 2.93 | Pliocene | 47.02 |
| TKS1-3 | Java | 2.93 | Pliocene | 43.86733 |
| TKS1-4 | Java | 2.93 | Pliocene | 19.02316 |
| TKS1-5 | Java | 2.93 | Pliocene | 17.61056 |
| TKS1-6 | Java | 2.93 | Pliocene | 47.22905 |
| LLD | Leyte | 2.605 | Pliocene | 89.61641 |
| S177 | Leyte | 2.605 | Pliocene | 89.52949 |
| S209 | Leyte | 2.605 | Pliocene | 87.12542 |
| TinagoC | Leyte | 2.46 | Pleistocene | 64.86824 |
| LLH | Leyte | 2.325 | Pleistocene | 87.75223 |
| S173 | Leyte | 2.155 | Pleistocene | 45.61927 |
| LLC | Leyte | 2.04 | Pleistocene | 79.70566 |
| LLE | Leyte | 2.04 | Pleistocene | 85.00909 |
| LLF | Leyte | 1.98 | Pleistocene | 82.97701 |
| S176 | Leyte | 1.905 | Pleistocene | 72.49892 |
| S183 | Leyte | 1.905 | Pleistocene | 81.11839 |
| S218 | Leyte | 1.905 | Pleistocene | 82.78962 |
| CRC2 | Cebu | 1.806 | Pleistocene | 43.59262 |
| S206 | Leyte | 1.675 | Pleistocene | 75.81172 |
| DVO11 | Mindanao | 1.675 | Pleistocene | 39.91689 |
| S200 | Leyte | 1.665 | Pleistocene | 99.63815 |
| LLG | Leyte | 1.615 | Pleistocene | 86.68108 |
| S170 | Leyte | 1.615 | Pleistocene | 57.14888 |
| S205 | Leyte | 1.615 | Pleistocene | 85.33739 |
| BOJ2B | Java | 1.296 | Pleistocene | 25.44181 |
| BOJ2C | Java | 1.296 | Pleistocene | 11.86458 |
| BOJ3 | Java | 1.296 | Pleistocene | 26.92514 |
| S181 | Leyte | 1.27 | Pleistocene | 50.82772 |
| Leyte26 | Leyte | 0.965 | Pleistocene | 52.53596 |
| TinagoD | Leyte | 0.53 | Pleistocene | 47.73296 |
| TinagoF | Leyte | 0.53 | Pleistocene | 55.24862 |

**Supplementary table 2.** **Bayesian estimation of the multivariate birth-death model (MBD) for the Cenozoic Era.** Posterior parameter estimates for the MBD model. Baseline speciation and extinction rates (λ0 and µ0) and correlation parameters (Gλ and Gµ). The environmental drivers are as follows: diversity of all ostracods through time, regional changes in coastline length through time, regional changes in total shelf area through time, global sea-level fluctuations through time, and global temperature changes through time. To be considered as having a significant effect, a variable must have a 95% highest posterior density (HPD) not overlap with 0 for G and a Shrinkage weights (ω) greater than 0.5 (highlighted in bold).

| **Parameters** | **mean** | **median** | **95% HPD** |
| --- | --- | --- | --- |
| λ0 | 0.4535 | 0.3015 | [0.0191, 1.355] |
| μ0 | 0.1807 | 0.1702 | [0.0175, 0.3519] |
| GλDiversity | **-2.5945** | **-2.7346** | **[-4.2589, -0.4659]** |
| GλCoastline | 0 | 0 | [-0.0001, 7.9403E-5] |
| GλSea level | 0.0123 | 0.0117 | [-0.0106, 0.0396] |
| GλShelf | **2.2673E-7** | **2.272E-7** | **[3.3637E-8, 4.1237E-7]** |
| GλTemperature | -0.0637 | -0.092 | [-0.5048, 0.472] |
| GμDiversity | 0.5444 | 0.3722 | [-0.5667, 2.1152] |
| GμCoastline | -0.0001 | 0 | [-0.0002, 3.7105E-5] |
| GμSea level | -0.0006 | -0.0001 | [-0.0144, 0.0118] |
| GμShelf | 0 | 0 | [-0, -0] |
| GμTemperature | 0.0542 | 0.026 | [-0.1076, 0.2797] |
| ωλDiversity | **0.7948** | **0.863** | **[0.3337, 1]** |
| ωλCoastline | 0.3562 | 0.2721 | [4.0384E-9, 0.9335] |
| ωλSea level | 0.577 | 0.642 | [0.0217, 1] |
| ωλShelf | **0.6144** | **0.6467** | **[0.1429, 1]** |
| ωλTemperature | 0.6744 | 0.7755 | [0.0636, 1] |
| ωμDiversity | 0.4234 | 0.3834 | [5.85E-9, 0.9547] |
| ωμCoastline | 0.4909 | 0.5066 | [9.5319E-9, 0.966] |
| ωμSea level | 0.3389 | 0.2381 | [1.4621E-9, 0.9295] |
| ωμShelf | 0.7929 | 0.8591 | [0.3504, 1] |
| ωμTemperature | 0.4047 | 0.3445 | [1.5743E-8, 0.9554] |

**Supplementary table 3.** **Bayesian estimation of the time slice MBD model for the warm period (23.04-13.9 Ma) of the Neogene.** All parameters as in Supplementary table 2.

| **Parameters** | **mean** | **median** | **95% HPD** |
| --- | --- | --- | --- |
| λ0 | 0.4252 | 0.2524 | [0.0873, 1.5727] |
| μ0 | 0.4828 | 0.3724 | [4.3802E-3, 1.2714] |
| GλDiversity | **25.1621** | **25.0933** | **[14.0198, 36.205]** |
| GλCoastline | -0.0001 | -0.0001 | [-0.0002, 7.285E-5] |
| GλSea level | **-0.0688** | **-0.0689** | **[-0.098, -0.0379]** |
| GλShelf | 0 | 0 | [-0, -0] |
| GλTemperature | **0.5746** | **0.5838** | **[0.199, 0.9292]** |
| GμDiversity | **328.7838** | **336.1681** | **[204.0052, 442.0327]** |
| GμCoastline | 6.4291E-3 | 6.9891E-3 | [4.8343E-4, 9.5262E-3] |
| GμSea level | **-0.8217** | **-0.8427** | **[-1.0903, -0.5165]** |
| GμShelf | 0 | 0 | [-0, -0] |
| GμTemperature | **4.994** | **5.0376** | **[3.422, 6.4536]** |
| ωλDiversity | **0.9981** | **0.999** | **[0.993, 1]** |
| ωλCoastline | 0.8191 | 0.9626 | [0.1095, 1] |
| ωλSea level | **0.9842** | **0.9947** | **[0.933, 1]** |
| ωλShelf | 0.9561 | 0.9892 | [0.797, 1] |
| ωλTemperature | **0.9726** | **0.9929** | **[0.8833, 1]** |
| ωμDiversity | **1** | **1** | **[0.9999, 1]** |
| ωμCoastline | 0.9974 | 0.9999 | [0.9958, 1] |
| ωμSea level | **0.9997** | **0.9998** | **[0.9989, 1]** |
| ωμShelf | 0.9999 | 0.9999 | [0.9996, 1] |
| ωμTemperature | **0.9992** | **0.9995** | **[0.9972, 1]** |

**Supplementary table 4.** **Bayesian estimation of the time slice MBD model for the cooling period (13.9-5.33 Ma) of the Neogene.** All parameters as in Supplementary table 2.

| **Parameters** | **mean** | **median** | **95% HPD** |
| --- | --- | --- | --- |
| λ0 | 0.5369 | 0.2359 | [0.0109, 1.6851] |
| μ0 | 0.2751 | 0.222 | [1.6396E-4, 0.6918] |
| GλDiversity | -0.0946 | -0.3026 | [-1.9147, 2.6444] |
| GλCoastline | 2.8726E-5 | 1.1733E-5 | [-0.0001, 1.5102E-4] |
| GλSea level | -0.0203 | -0.0183 | [-0.0441, 1.1104E-4] |
| GλShelf | 0 | 0 | [-0, 3.8466E-8] |
| GλTemperature | 0.2191 | 0.1374 | [-0.0859, 0.7968] |
| GμDiversity | 1.8362 | 1.7161 | [-0.1831, 4.1565] |
| GμCoastline | 2.5164E-5 | 5.0431E-6 | [-0.0001, 2.2481E-4] |
| GμSea level | -0.0152 | -0.0123 | [-0.0456, 5.2491E-3] |
| GμShelf | 0 | 0 | [-0, -0] |
| GμTemperature | 0.1023 | 0.0301 | [-0.1782, 0.5573] |
| ωλDiversity | 0.541 | 0.5884 | [7.2072E-9, 0.9763] |
| ωλCoastline | 0.4102 | 0.3467 | [7.6009E-9, 0.9625] |
| ωλSea level | 0.7069 | 0.7842 | [0.1744, 1] |
| ωλShelf | 0.5403 | 0.571 | [1.7734E-9, 0.9716] |
| ωλTemperature | 0.6094 | 0.7098 | [0.0146, 1] |
| ωμDiversity | 0.6925 | 0.7841 | [0.0897, 1] |
| ωμCoastline | 0.4512 | 0.424 | [8.8184E-11, 0.9717] |
| ωμSea level | 0.5889 | 0.6714 | [0.0152, 1] |
| ωμShelf | 0.9039 | 0.9384 | [0.6934, 1] |
| ωμTemperature | 0.4965 | 0.5067 | [1.7672E-8, 0.9832] |

**Supplementary table 5.** **Bayesian estimation of the time slice MBD model for the cold period (5.33-0 Ma) of the Neogene-Quaternary.** All parameters as in Supplementary table 2.

| **Parameters** | **mean** | **median** | **95% HPD** |
| --- | --- | --- | --- |
| λ0 | 0.7037 | 0.5289 | [0.1164, 1.3337] |
| μ0 | 0.1708 | 0.159 | [0.0186, 0.3403] |
| GλDiversity | **-3.301** | **-3.3412** | **[-4.3557, -1.9571]** |
| GλCoastline | 0 | 0 | [-0.0001, 3.3972E-5] |
| GλSea level | **0.0225** | **0.0223** | **[3.0546E-3, 0.0419]** |
| GλShelf | **2.5015E-7** | **2.4447E-7** | **[7.5419E-8, 4.3238E-7]** |
| GλTemperature | -0.2661 | -0.2786 | [-0.5438, 0.0977] |
| GμDiversity | 0.6212 | 0.4508 | [-0.5467, 2.2779] |
| GμCoastline | -0.0001 | -0.0001 | [-0.0002, 3.8859E-5] |
| GμSea level | -0.0005 | 0 | [-0.0146, 0.0124] |
| GμShelf | 0 | 0 | [-0, -0] |
| GμTemperature | 0.0611 | 0.0335 | [-0.107, 0.2918] |
| ωλDiversity | **0.8775** | **0.9116** | **[0.643, 1]** |
| ωλCoastline | 0.3945 | 0.3319 | [1.7298E-11, 0.9496] |
| ωλSea level | **0.7411** | **0.8188** | **[0.2187, 1]** |
| ωλShelf | **0.6599** | **0.702** | **[0.2057, 1]** |
| ωλTemperature | 0.7513 | 0.8481 | [0.1755, 1] |
| ωμDiversity | 0.4649 | 0.456 | [1.1205E-9, 0.9663] |
| ωμCoastline | 0.5197 | 0.5584 | [2.5186E-9, 0.9713] |
| ωμSea level | 0.3687 | 0.2836 | [2.3212E-10, 0.9444] |
| ωμShelf | 0.8006 | 0.8653 | [0.3732, 1] |
| ωμTemperature | 0.4423 | 0.4107 | [3.1265E-9, 0.9652] |

**Supplementary table 6.** **Bayesian estimation of the MBD model for the Cenozoic Era with the tropical temperature record from Scotese, et al ^77^.** All other parameters as in Supplementary table 2.

| **Parameters** | **mean** | **median** | **95% HPD** |
| --- | --- | --- | --- |
| λ0 | 0.3128 | 0.1256 | [0.0463, 1.1074] |
| μ0 | 0.2053 | 0.196 | [0.0575, 0.3596] |
| GλDiversity | **-2.4091** | **-2.3445** | **[-3.2315, -1.71]** |
| GλCoastline | 5.19E-06 | 2.3E-06 | [-0, 5.5052E-5] |
| GλSea level | 0.006017 | 0.00637 | [-0.0011, 0.0126] |
| GλShelf | **2.05E-07** | **2.1E-07** | **[4.1919E-8, 3.5623E-7]** |
| GλTemperature | 0.0396 | 0.0576 | [-0.2314, 0.2679] |
| GμDiversity | 0.3169 | 0.1995 | [-0.5891, 1.46] |
| GμCoastline | -0.0001 | 0 | [-0.0002, 3.6475E-5] |
| GμSea level | 0.001371 | 0.00065 | [-0.0066, 0.0108] |
| GμShelf | 0 | 0 | [-0, -0] |
| GμTemperature | 0.0131 | 0.00342 | [-0.0901, 0.1383] |
| ωλDiversity | **0.811** | **0.8469** | **[0.5129, 1]** |
| ωλCoastline | 0.2702 | 0.1526 | [8.6326E-10, 0.8823] |
| ωλSea level | 0.4236 | 0.3844 | [1.6839E-8, 0.9345] |
| ωλShelf | **0.5847** | **0.6029** | **[0.1309, 1]** |
| ωλTemperature | 0.709 | 0.8221 | [0.0705, 1] |
| ωμDiversity | 0.3535 | 0.2704 | [9.2322E-10, 0.9268] |
| ωμCoastline | 0.4839 | 0.4977 | [7.0011E-10, 0.9638] |
| ωμSea level | 0.292 | 0.179 | [2.0434E-9, 0.8979] |
| ωμShelf | 0.7877 | 0.8514 | [0.3489, 1] |
| ωμTemperature | 0.3953 | 0.3275 | [2.1195E-9, 0.9499] |

**Supplementary table 7.** **Bayesian estimation of the MBD model for the Neogene-Quaternary with the tropical temperature record from Scotese, et al ^77^.** All other parameters as in Supplementary table 2.

| **Parameters** | **mean** | **median** | **95% HPD** |
| --- | --- | --- | --- |
| λ0 | 0.6232 | 0.6738 | [5.9642E-3, 1.5873] |
| μ0 | 0.2075 | 0.1986 | [0.063, 0.3659] |
| GλDiversity | **-2.6354** | **-2.7686** | **[-3.4045, -1.167]** |
| GλCoastline | 7.47E-06 | 4.1E-06 | [-0, 6.1824E-5] |
| GλSea level | 0.003336 | 0.00217 | [-0.0033, 0.013] |
| GλShelf | **2.19E-07** | **2.2E-07** | **[5.6876E-8, 3.6652E-7]** |
| GλTemperature | -0.0391 | -0.12 | [-0.2785, 0.5748] |
| GμDiversity | 0.3151 | 0.1932 | [-0.5964, 1.456] |
| GμCoastline | -0.0001 | 0 | [-0.0002, 3.8574E-5] |
| GμSea level | 0.001072 | 0.00047 | [-0.0068, 0.0104] |
| GμShelf | 0 | 0 | [-0, -0] |
| GμTemperature | 0.0134 | 0.00359 | [-0.092, 0.1367] |
| ωλDiversity | **0.8241** | **0.8638** | **[0.5211, 1]** |
| ωλCoastline | 0.276 | 0.159 | [7.136E-11, 0.8852] |
| ωλSea level | 0.3207 | 0.2209 | [2.717E-9, 0.9103] |
| ωλShelf | **0.5987** | **0.6213** | **[0.1506, 1]** |
| ωλTemperature | 0.7315 | 0.8524 | [0.0487, 1] |
| ωμDiversity | 0.3498 | 0.2644 | [3.0755E-9, 0.9263] |
| ωμCoastline | 0.4745 | 0.4819 | [2.5487E-8, 0.9612] |
| ωμSea level | 0.2849 | 0.171 | [6.0794E-10, 0.8911] |
| ωμShelf | 0.7869 | 0.8507 | [0.3476, 1] |
| ωμTemperature | 0.3908 | 0.3207 | [2.9372E-9, 0.9487] |
